# Mechanism of exogenous methyl jasmonate in regulating the quality of fresh-cut Chinese water chestnuts

**DOI:** 10.3389/fpls.2024.1435066

**Published:** 2024-08-15

**Authors:** Keyan Lu, Xinping Wu, Ruimin Yuan, Yang Yi, Limei Wang, Youwei Ai, Hongxun Wang, Ting Min

**Affiliations:** ^1^ College of Food Science and Engineering, Wuhan Polytechnic University, Wuhan, China; ^2^ Hubei Key Laboratory for Processing and Transformation of Agricultural Products (Wuhan Polytechnic University), Wuhan, China; ^3^ School Biology and Pharmaceutical Engineering, Wuhan Polytechnic University, Wuhan, China

**Keywords:** fresh-cut, Chinese water chestnut, methyl jasmonate, yellowing, quality

## Abstract

Fresh-cut Chinese water chestnuts (CWCs) are susceptible to yellowing and browning during storage due to mechanical damage and the loss of protective outer skin, adversely affecting their marketability and shelf life. Methyl jasmonate (MeJA) is currently extensively used for food preservation, but it has not been used in Chinese water chestnuts. This study investigated the effect of MeJA treatment on the quality of fresh-cut CWCs. Fresh-cut CWCs immersed in 20 μM MeJA solution for 10 min and stored at 10°C for 5 d effectively delayed the yellowing process, reduced the respiration rate, and minimized the weight and soluble solids loss during storage. In addition, MeJA treatment induced the activities of superoxide dismutase (SOD) and catalase (CAT), which improved the antioxidant capacity of fresh-cut CWCs and inhibited the generation of reactive oxygen species (ROS). Meanwhile, MeJA treatment inhibited the activities of phenylalanine aminotransferase (PAL), polyphenol oxidase (PPO) and peroxidase (POD). The results of quantitative real-time PCR (qRT-PCR) showed that MeJA down-regulated the expression of *CwCHS1*, *CwCHS2*, *CwCHS3* and *CwCHI2* in freshly cut CWCs and inhibited the accumulation of flavonoids, thus delaying the surface discoloration of freshly cut CWCs.

## 1 Introduction

Chinese water chestnut (CWC, Eleocharis Tuberosa) is a commonly found aquatic plant known for its edible bulb with a sweet, juicy, and crisp texture, which is highly popular in China (Song et al., 2019). However, CWC bulbs tend to be covered in muddy outer purplish-brown skin, necessitating cleaning and peeling before consumption. Manual cleaning and peeling processes are time-consuming and escalate labor costs. To address these challenges and provide convenience to consumers, the industry has introduced sorting machines, cleaning machines, and peelers (Zhou et al., 2022) to achieve standardized processing of fresh-cut CWCs and enhance production efficiency. Despite these advancements, removing the outer skin during processing exposes the meat of fresh-cut CWCs to mechanical damage, rendering its cells vulnerable. Consequently, fresh-cut CWCs are highly susceptible to yellowing during storage, leading to a rapid decline in organoleptic quality and a loss of commercial value (Li et al., 2022).

The phenomenon of browning, which occurs in many freshly cut fruits and vegetables, primarily results from enzymatic activity (Hasan et al., 2020). In the presence of oxygen, phenolic compounds in these produce items are transformed into quinones through the action of PPO and POD. These quinones polymerize into brown pigments (Teng et al., 2020). Additionally, the presence of excessive ROS, such as superoxide anion (O_2_·^-^) and hydrogen peroxide (H_2_O_2_), accelerates browning and can lead to membrane lipid peroxidation, causing increased permeability (Zhu et al., 2022a). The disruption of cell membrane integrity accelerates the reaction of phenolics and other substances with oxygen and enzymes, resulting in the rapid deterioration of the appearance of fruits and vegetables (Zha et al., 2022).

As described by previous authors, discoloration of fresh-cut CWCs is primarily due to specific metabolites on the surface, with yellowing substances mainly identified as flavonoids, such as eriodictyol and naringenin (Pan et al., 2015). The synthesis of flavonoids involves key enzymes in the phenylpropanoid pathway, such as PAL, chalcone isomerase (CHI), and chalcone synthase (CHS) (Li et al., 2022). It is now understood that PAL serves as a bridge between primary and phenylpropanoid metabolism, initiating the catalysis of phenylpropanoid metabolism. This metabolic pathway generates secondary compounds like phenols, lignin, and flavonoids, which are susceptible to enzymatic browning and can transform into brown substances under the influence of PPO and POD (Teng et al., 2020).

Much research has been conducted to retard the quality deterioration of fresh-cut CWCs. Early approaches involved chitosan coating (Pen and Jiang, 2003), citric acid (Jiang et al., 2004), and hydrogen peroxide (Peng et al., 2008) to inhibit yellowing. In consideration of environmental friendliness and food safety, more recent studies have explored soaking treatments with ascorbic acid and ferulic acid (Song et al., 2019), hydrogen sulfide (Dou et al., 2021), melatonin (Xu et al., 2022), and hydrogen-rich water (Li et al., 2022). We are also investigating convenient postharvest techniques to inhibit the yellowing of fresh-cut CWCs. A literature review revealed that methyl jasmonate, a naturally occurring plant compound, is currently under investigation for food preservation (Wang et al., 2021).

Methyl jasmonate (MeJA) is a volatile phytohormone with robust biological activity (Cheong and Do Choi, 2003). As a signaling molecule, MeJA plays a pivotal role in various physiological and biochemical processes in plants and regulates the synthesis of other hormones (Per et al., 2018). While storing numerous postharvest fruits and vegetables, MeJA has been shown to maintain quality and enhance systemic acquired resistance (Wang et al., 2021). Studies have demonstrated that exogenous MeJA increases the production of volatiles, phenolics, and unsaturated fatty acids in postharvest fruits and vegetables. MeJA promotes the release of aroma-related lactones in peaches (Cai et al., 2020), significantly elevates carotenoid content in cherry tomatoes after harvest (Liu et al., 2018b), induces the synthesis of ripening aromatic volatiles (Qin et al., 2017), enhances ester synthesis capacity in Nanguo pears (Luo et al., 2021), and improves the flavor quality of postharvest fruits. MeJA also contributes to color and firmness improvement, delays aging, and reduces or prevents cold damage symptoms by boosting antioxidant enzyme activity and promoting antioxidant production in postharvest fruits and vegetables (Dong et al., 2016; Wang et al., 2019b; Zhu et al., 2022b). Concha et al. (2013) found that MeJA promotes fragaria chiloensis fruit ripening and defense-related processes through up-regulation of anthocyanin-related genes (*CHS*, *CHI*, *F3H*). In addition, ethephon and 1-methylcyclopropene were found to inhibit flavonoid accumulation in fresh-cut CWCs by down-regulating *CwCHS1* and *CwCHI1* expression in fruit (Xu et al., 2023b). However, MeJA has hitherto not been applied to fresh-cut CWCs preservation.

Given the various factors contributing to the yellowing of fresh-cut CWCs, including enzymatic browning, active oxygen metabolism, membrane lipid metabolism, and flavonoid accumulation (Pan et al., 2015; Zhu et al., 2022a), we hypothesized that MeJA may delay changes in the appearance of fresh-cut CWCs through these pathways. Consequently, the aim of our study was to assess the effect of MeJA on fresh-cut CWCs and its mechanisms by measuring basic quality indicators, relevant indicators of antioxidant system and reactive oxygen species metabolism, and expression of genes related to the phenylpropane metabolic pathway. We also provided insights that may prove valuable for the application of MeJA in preserving the quality of other food products.

## 2 Materials and methods

### 2.1 Materials and treatment

CWCs were procured from a local market and pre-cooled at 4°C for 24 h. The laboratory was sterilized with ozone under completely closed conditions for two hours before commencing the experiment. Following washing and peeling, fresh CWCs with intact, thick, hard, and dark brown peels, measuring 35–45 cm in diameter and devoid of external damage, internal diseases, or pests, were selected for subsequent experiments. Based on prior experiments, we established an immersion duration of 10 minutes (Xu et al., 2020; Duan et al., 2022). We subsequently conducted experiments to determine the optimal MeJA concentration. Fresh-cut CWCs were immersed in MeJA solutions at 10, 20, 50, and 100 μM for 10 min, with 20 μM MeJA yielding superior inhibition of yellowing compared to other groups. Subsequent experiments were conducted in accordance with this result.

The selected CWCs were randomly divided into treatment and control groups after immersing them in a 0.1% NaClO solution for 5 minutes. The treatment group was immersed in a 20 μM MeJA solution (containing 1% anhydrous ethanol for dissolution), while the control group was immersed in distilled water containing 1% anhydrous ethanol. After 10 min of immersion, all CWCs were removed to allow drying. Each group of two CWCs was sealed in a polyethylene bag (200 × 280 mm) containing a polyethylene tray (180 × 120 × 25 mm) (Xu et al., 2023a). Subsequently, all samples were stored at 10°C and analyzed daily. The sample tissues were frozen in liquid nitrogen and stored in -80°C for backup.

### 2.2 Appearance and degree of browning

Camera photography was employed to assess the appearance of fresh-cut CWCs using images (Canon, EOS550D). The L*, a*, and b* values were determined using a JZ-300 colorimeter (Shenzhen Jinzhun Instrument Equipment Co., Ltd., China). The color difference (,E) was calculated using the following equation:


$$\Delta \text{E}=\sqrt{{\left(L^{*}-L_{0}^{*}\right)}^{2}+{\left(a^{*}-a_{0}^{*}\right)}^{2}+{\left(b^{*}-b_{0}^{*}\right)}^{2}}$$

While L_0_, a_0_, and b_0_ were all values on the 0th day, L*, a*, and b* were readings at each sampling point during the storage period. Measurement of the degree of browning was in accordance with the method of Min et al. (2017) and was expressed as A_410 nm_ ×10.

### 2.3 Weight loss rate, total soluble solids content, O_2_ and CO_2_ content in bags

Weight loss rates of fresh-cut CWCs were evaluated using the weighing method as described by Wu et al. (2024). The measurement of total soluble solid content was referenced from a study by Xu et al. (2022). Tissues weighing 10 g were manually ground and filtered through a fine cotton gauze. Subsequently, total soluble solids were assessed using a portable refractometer (Wu et al., 2024).

According to Wu et al. (2024), O_2_ and CO_2_ contents in bags of fresh-cut CWCs were determined using a portable headspace analyzer (Checkpoint 3, Mocon, Denmark).

### 2.4 Total phenolics, total flavonoid, and soluble quinone content

The total phenolics content (TPC) was determined using the method of Min et al. (2017), and the results were quantified with standard gallic acid samples, presented in mg·kg^-1^. The determination of soluble quinone and total flavonoid content (TFC) followed the procedure outlined by Xu et al. (2022). Their absorbance was measured at 437 nm and 510 nm, and the results were presented as A_437 nm_·g^-1^ and mg·kg^-1^, respectively.

### 2.5 PAL, PPO, and POD activities

PAL, PPO and POD activities were assessed according to previous descriptions (Min et al., 2019). The variation of PAL, PPO and POD activities were measured at 290, 420 and 470 nm per minute. Defined as the amount of enzyme required per gram of fresh weight for a change in absorbance value (0.1, 0.001 and 0.01), respectively, the results were expressed as U•g^-1^.

### 2.6 O_2_•^-^ and OH•^-^ generation rate, H_2_O_2_ and malondialdehyde (MDA) content

O_2_·^-^ generation rate was performed as described by Chen et al. (2022), and the results are rendered in nmol·g^-1^·min^-1^. OH·^-^ generation rate and H_2_O_2_ content were evaluated using OH·^-^ and H_2_O_2_ kits (Nanjing Jianjian Bioengineering Research Institute Co., Ltd., Nanjing, China), and the results are presented in mmol·g^-1^·min^-1^ and mmol·g^-1^, respectively. MDA content was measured in accordance with Xu et al. (2022) and expressed in μmol·g^-1^.

### 2.7 SOD and CAT activities

Based on the description of Chen et al. (2022), changes in activity were evaluated using SOD and CAT kits (Nanjing Jianjian Bioengineering Research Institute Co., Ltd., Nanjing, China), and the results are rendered in U•g^-1^.

### 2.8 Expression of genes related to phenylpropane metabolic pathway

According to our previous study, the sequences of genes encoding key enzymes of the phenylpropanoid pathway were obtained based on the NCBI database (*CwCHS1、CwCHS2、CwCHS3、CwCHI2*) (Xu et al., 2023b). Extraction of RNA from CWCs and cDNA synthesis and qRT-PCR reactions were performed as described by Xu et al. (2023b). Three biological replicates were performed for each sampling site. The internal reference gene in this study was *CwActin* (MG742687.1). Primer sequences were designed using Primer 5.0 software (**Supplementary Table 1**).

### 2.9 Statistical analysis

The experiment was repeated three times and the results were expressed as mean ± standard error. Comparisons of means between groups were analyzed by one way analysis of variance (ANOVA) using SPSS 19 followed by Duncan’s test. *p* < 0.05 indicates statistical significance.

## 3 Results

### 3.1 The effect of MeJA on appearance, color change, and browning degree

**Figure 1:**
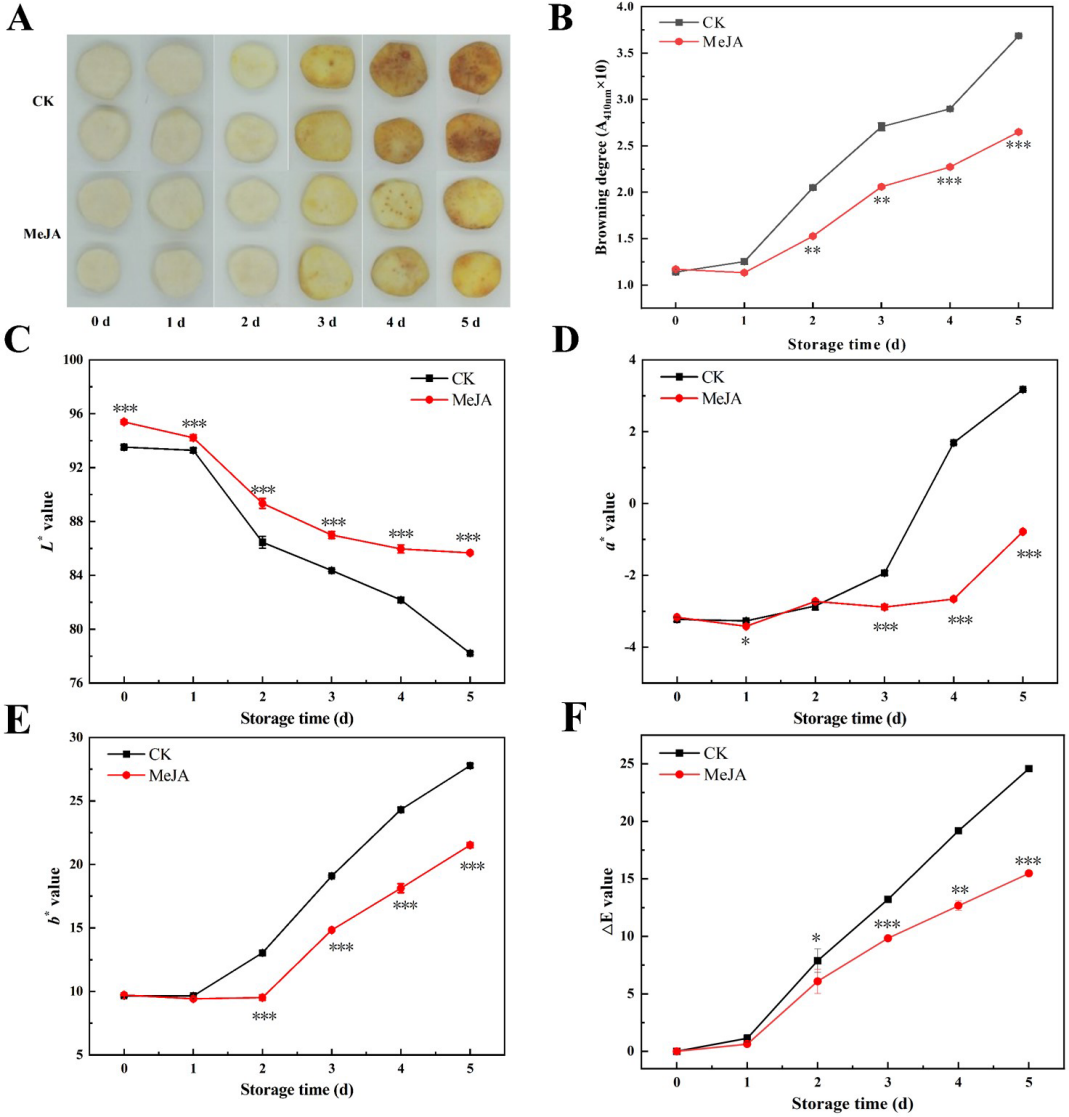
The effect of MeJA treatment on the appearance **(A)**, browning degree **(B)**, color change **(C-E)**, and ,ΔE value **(F)** of fresh-cut CWCs. Error bars represent the standard error of three biological replicates. *、**、***: represent the level of difference between MeJA group and CK group is *p* < 0.05、*p* < 0.01、*p* < 0.001, respectively.

Appearance quality and color of fruits are critical factors in assessing its quality (Zhu et al., 2022b) and significantly influence consumer purchasing decisions. As shown in **Figure 1A**, fresh-cut CWCs exhibited significant yellowing during storage. The CWCs in the control group displayed pronounced yellowing on the third day, while CWCs soaked in MeJA exhibited less discoloration. Severe yellowing appeared on the surface of the control group in the last two days, whereas discoloration in the MeJA-treated group was significantly inhibited. As shown in **Figure 1B**, the browning degree of fresh-cut CWCs gradually increased with time. On day 5, the browning degree increased 2.26-fold in the MeJA group and 3.24-fold in the CK group compared to day 0. The MeJA group consistently exhibited significantly less browning than the control group.

The color difference values reflect the color change of fresh-cut CWCs in numerical form (Wu et al., 2024). The L* value of fresh-cut CWCs decreased continuously during storage, but the MeJA-treated group remained consistently higher than the control group (*p* < 0.05) (**Figure 1C**). As shown in **Figures 1D, E**, the a* and b* values of CWCs increased continuously, and those of the MeJA group were significantly lower than the control group in the last three days. Compared to the control, MeJA significantly suppressed the decrease in L* values as well as the increase in a* and b* values of fresh-cut CWCs. During storage, the ,E value of the MeJA treatment group was always lower than that of the CK group, with an overall increasing trend, which was consistent with the appearance and browning results (**Figure 1F**). The results indicated that MeJA treatment could effectively delay the browning of fresh-cut CWCs.

### 3.2 The effect of MeJA on soluble solids content, weight loss rate, and headspace gas composition in bags

**Figure 2:**
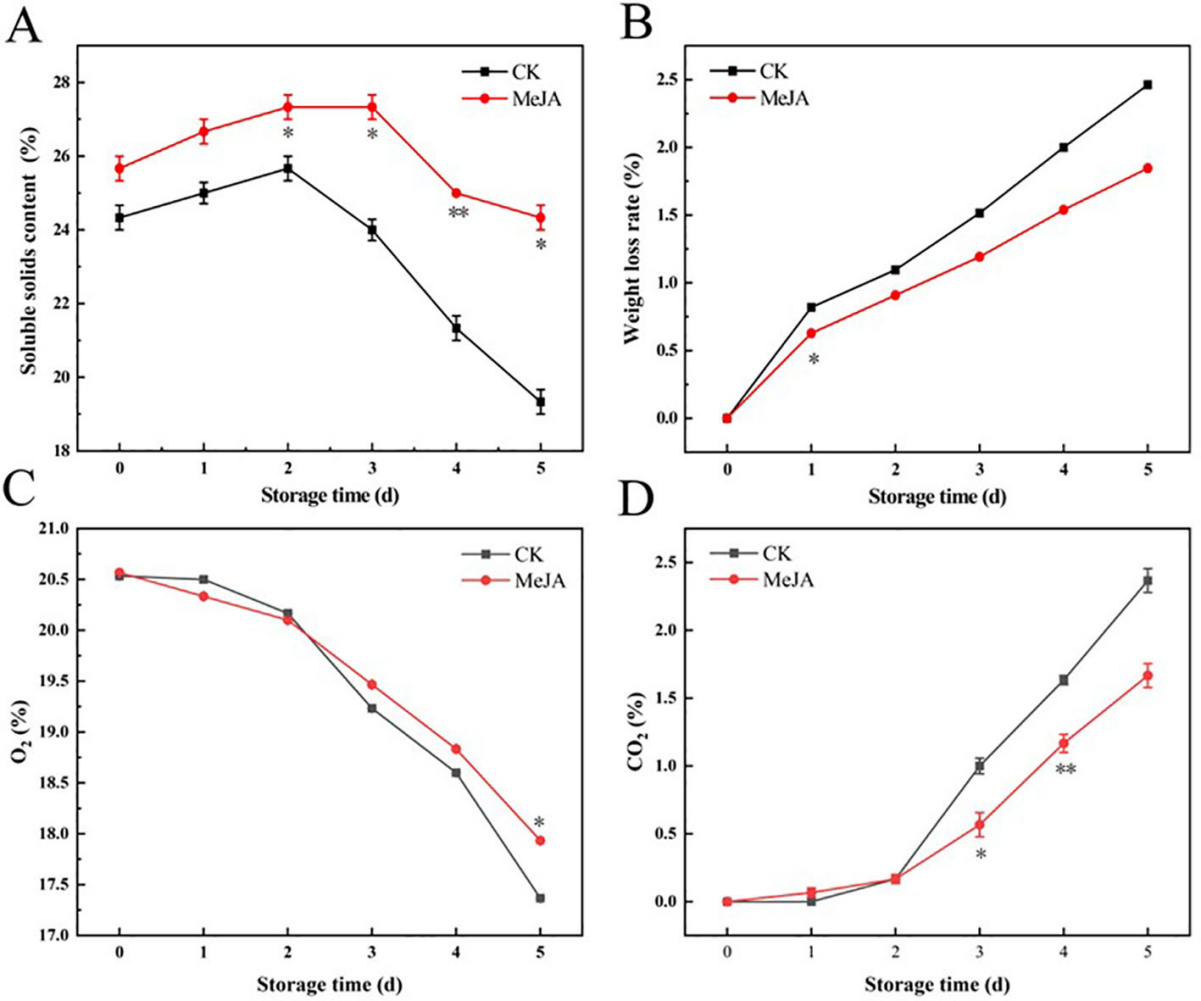
The effect of MeJA treatment on the soluble solids content **(A)**, weight loss rate **(B)**, O_2_ content **(C)** and CO_2_ content **(D)** of fresh-cut CWCs. Error bars represent the standard error of three biological replicates. *、**、***: represent the level of difference between MeJA group and CK group is *p* < 0.05、*p* < 0.01、*p* < 0.001, respectively.

The transition from fruit ripening to aging is often accompanied by decreased soluble solids content (Wu et al., 2024). As shown in **Figure 2A**, the soluble solid content initially increased and then decreased. The CK group reached its peak on day 2 and then experienced a sharp decline, while fresh-cut water chestnuts treated with MeJA soaking peaked on the third day. On day 5, the soluble solid content of the MeJA group decreased by 5.20% compared to the initial value, while that of the CK group decreased by 20.55%. MeJA resulted in a smoother and delayed decrease in the soluble solid content of CWCs compared to the control. The weight loss rate continuously increased, as depicted in **Figure 2B**, but MeJA suppressed this trend compared to the CK group. In summary, MeJA could effectively inhibit the reduction of weight and soluble solids in fresh-cut CWCs.

The headspace gas in the bags can indirectly reflect the respiration intensity of fresh-cut CWCs (Chen et al., 2022). Fresh-cut CWCs showed a gradual decrease in O_2_ content and a gradual increase in CO_2_ content (**Figures 2C, D**). Starting from day 3, MeJA effectively suppressed this change compared to the control, indicating that MeJA could effectively inhibit the respiration of fresh-cut CWCs.

### 3.3 The effect of MeJA on TPC, soluble quinone content, and TFC

**Figure 3:**
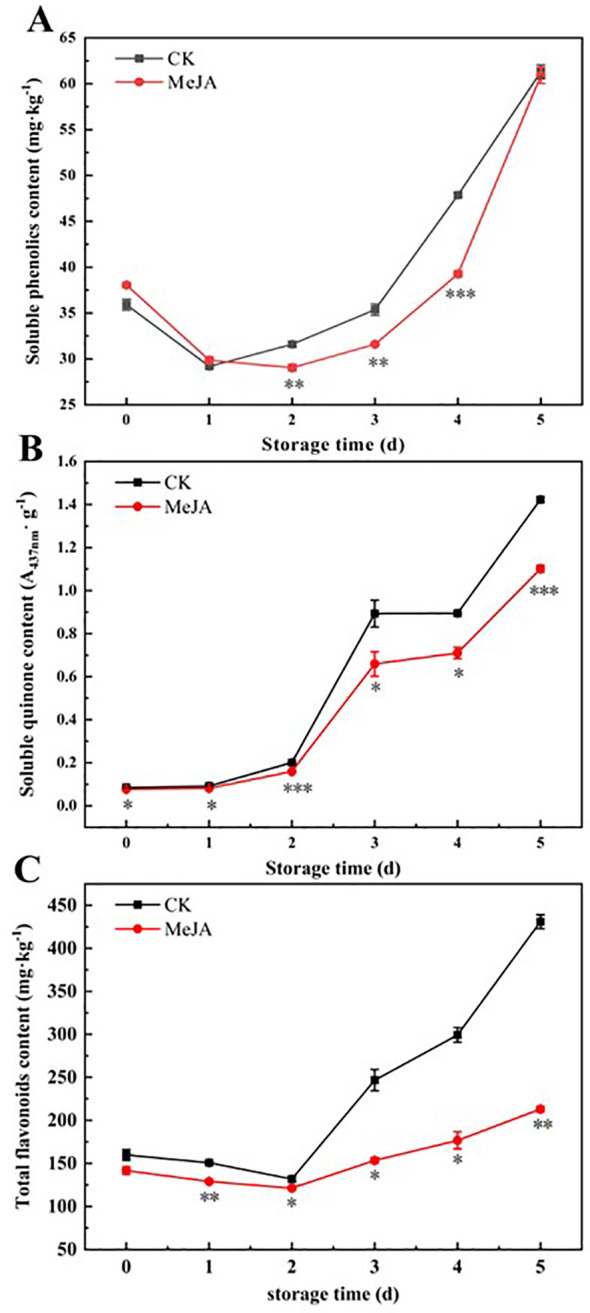
The effect of MeJA treatment on the soluble phenolics content **(A)**, soluble quinone content **(B)**, and total flavonoid content **(C)** of fresh-cut CWCs. Error bars represent the standard error of three biological replicates. *、**、***: represent the level of difference between MeJA group and CK group is *p* < 0.05、*p* < 0.01、*p* < 0.001, respectively.

Phenolic compounds can be converted to quinones through reactions catalyzed by PPO and POD in the presence of oxygen, forming brown pigments (Teng et al., 2020). Both TPC and soluble quinone contents of freshly cut CWCs exhibited an increasing trend, but those of the MeJA group increased to a lesser extent than the control (**Figures 3A, B**).

Pan et al. (2015) proposed that the yellowing of fresh-cut CWCs was due to the accumulation of flavonoids. In **Figure 3C**, the TFC of freshly cut CWCs increased rapidly with time. On day 5, TFC in the CK group increased by 69.44% compared to day 0, while the MeJA-treated group had only increased by 50.71%. MeJA significantly inhibited the increase in TFC compared to the control (*p* < 0.05). In conclusion, MeJA could delay the surface yellowing of fresh-cut CWCs by reducing the accumulation of soluble quinone and TFC.

### 3.4 The effect of MeJA on PAL, PPO, and POD activities

**Figure 4:**
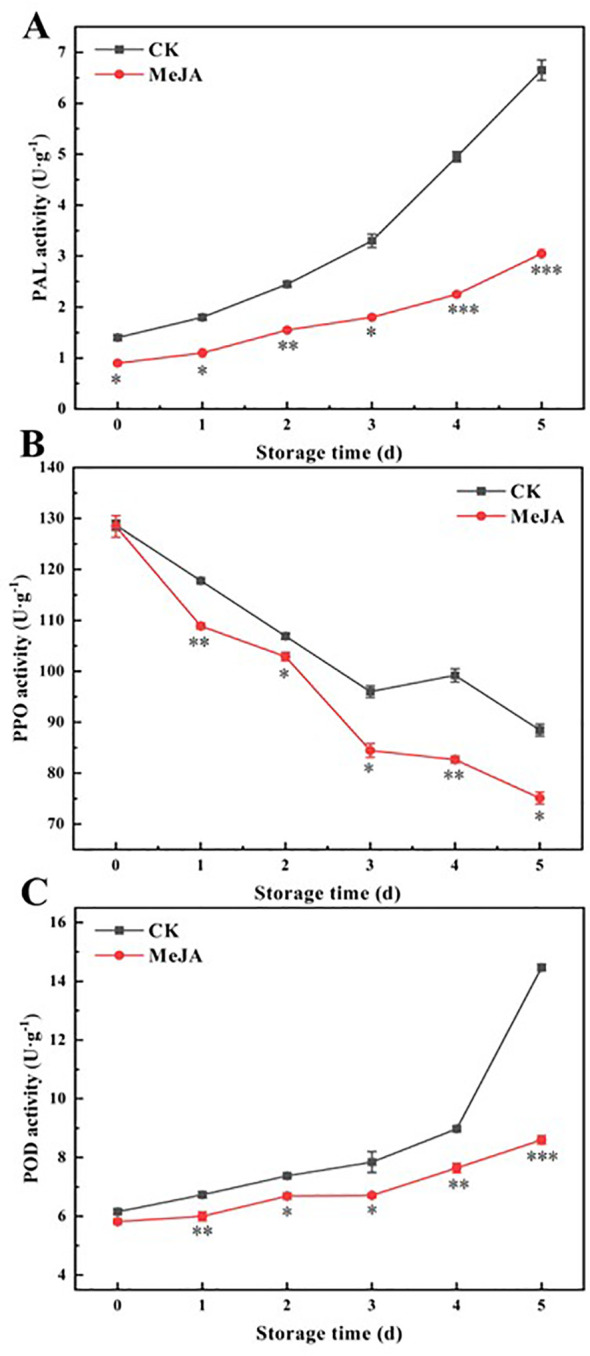
The effect of MeJA treatment on the activities of PAL **(A)**, PPO **(B)**, and POD **(C)** of fresh-cut CWCs. Error bars represent the standard error of three biological replicates. *、**、***: represent the level of difference between MeJA group and CK group is *p* < 0.05、*p* < 0.01、*p* < 0.001, respectively.

When plants experience mechanical damage, the activity of PAL in their tissues increases, enabling plants to produce more phenolics (Min et al., 2017). These phenolics react with O_2_ to produce quinones, catalyzed by PPO and POD, leading to the browning of fruits and vegetables (Liu et al., 2018a). According to **Figures 4A–C**, PAL and POD activities showed an increasing trend, while PPO activities continued to decrease. On day 5, PAL and POD activity in the control group increased by 4.75-fold and 2.35-fold compared to the initial values, while in the MeJA group, they increased by only 3.39-fold and 1.48-fold. Except for day 0, MeJA consistently and significantly maintained PAL, PPO, and POD activities at lower levels than the control throughout this period.

### 3.5 The effect of MeJA on O_2_^•-^ and OH^•-^ generation rate, H_2_O_2_ and malondialdehyde (MDA) content

An increasing body of evidence suggests that abiotic stress, such as mechanical injury or cold, induces the production of ROS in plants, including O_2_·^-^, OH·^-^, H_2_O_2_, and lipid peroxides, disrupting metabolic homeostasis and potentially causing oxidative stress an·d cell damage (Xu et al., 2023a). Excess ROS can increase cell membrane permeability, lipid peroxidation, and DNA mutation, resulting in oxidative stress and cell damage (Apel and Hirt, 2004). Oxidative stress can stimulate the biosynthesis of flavonoids (Li et al., 2022), further promoting the yellowing of fresh-cut CWCs.

**Figure 5:**
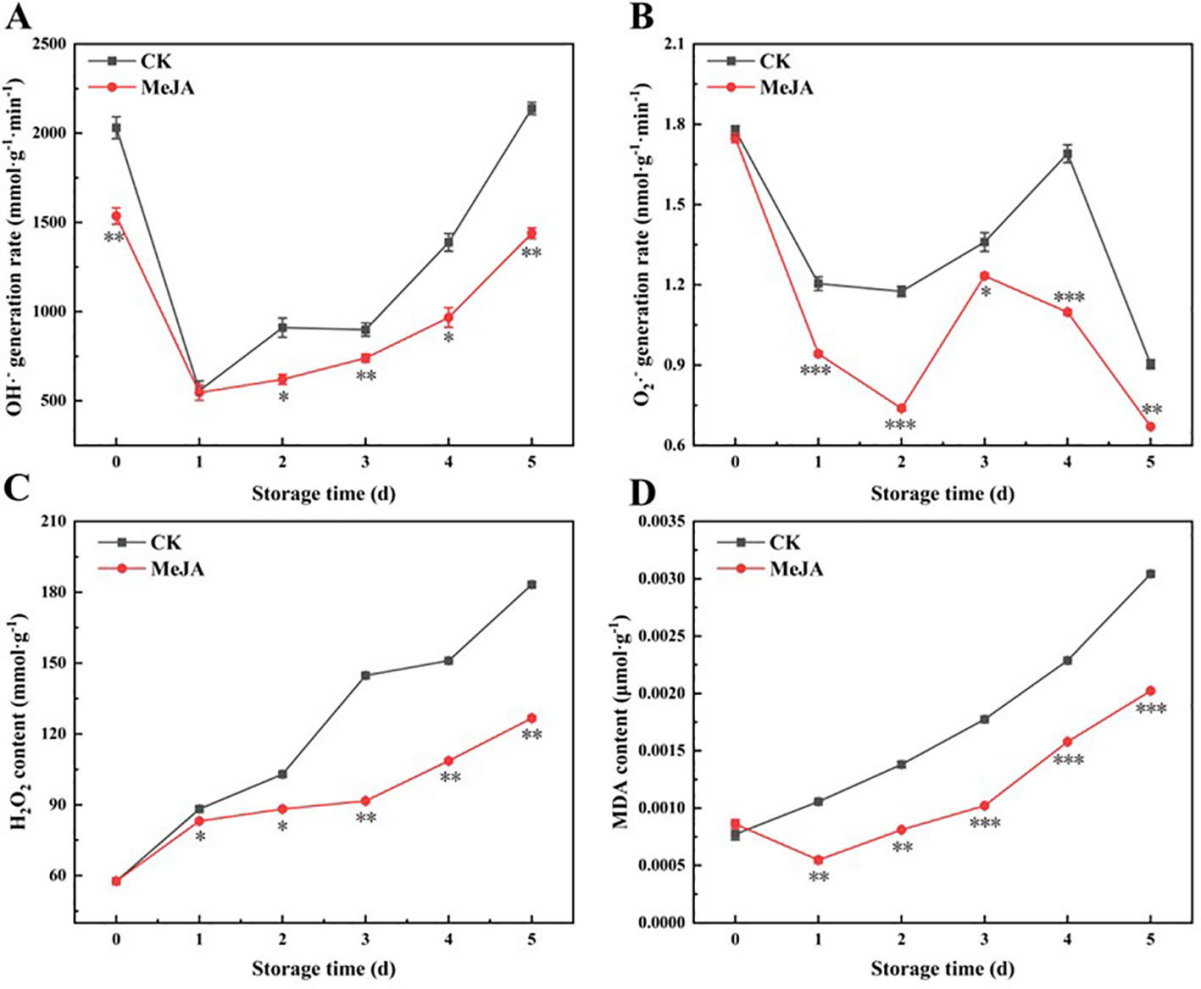
The effect of MeJA treatment on the OH·^-^ generation rate **(A)**, O_2_·^-^ generation rate **(B)**, H_2_O_2_ content **(C)** and MDA content **(D)** of fresh-cut CWCs. Error bars represent the standard error of three biological replicates. *、**、***: represent the level of difference between MeJA group and CK group is *p* < 0.05、*p* < 0.01、*p* < 0.001, respectively.

As shown in **Figure 5A**, the OH·^-^ production rate reached its peak or sub-peak on the first day due to mechanical damage. The production rate of both groups decreased substantially on the first day and reached essentially the same level. From the first day, the OH·^-^ production rate increased sharply in both treatment groups, although it consistently remained significantly lower in the MeJA group than the CK group. Both groups exhibited a fluctuating decrease in the O_2_·^-^ generation rate (**Figure 5B**). From day 0 to day 2, the O_2_·^-^ generation rate decreased continuously and then increased. The MeJA group began to decline on the third day, while the CK group began to decline after peaking on the fourth day. MeJA significantly inhibited the O_2_·^-^ generation rate compared to the control.

The H_2_O_2_ content increased with time (**Figure 5C**). Ultimately, the H_2_O_2_ content in the MeJA group was 2.20-fold higher than on day 0, whereas that in the control group was 3.18-fold higher. MeJA significantly inhibited the accumulation of H_2_O_2_ from the first day. MDA is used to characterize the extent of oxidative damage to cell membranes (Wu et al., 2024). The MDA content of fresh-cut CWCs increased during storage (**Figure 5D**). MeJA significantly inhibited the accumulation of MDA in fresh-cut CWCs during storage, except on the first day. At the end of storage (5d), the MDA content of the CK group was 1.50 times higher as compared to the MeJA-treated group.

### 3.6 The effect of MeJA on SOD and CAT activities

**Figure 6:**
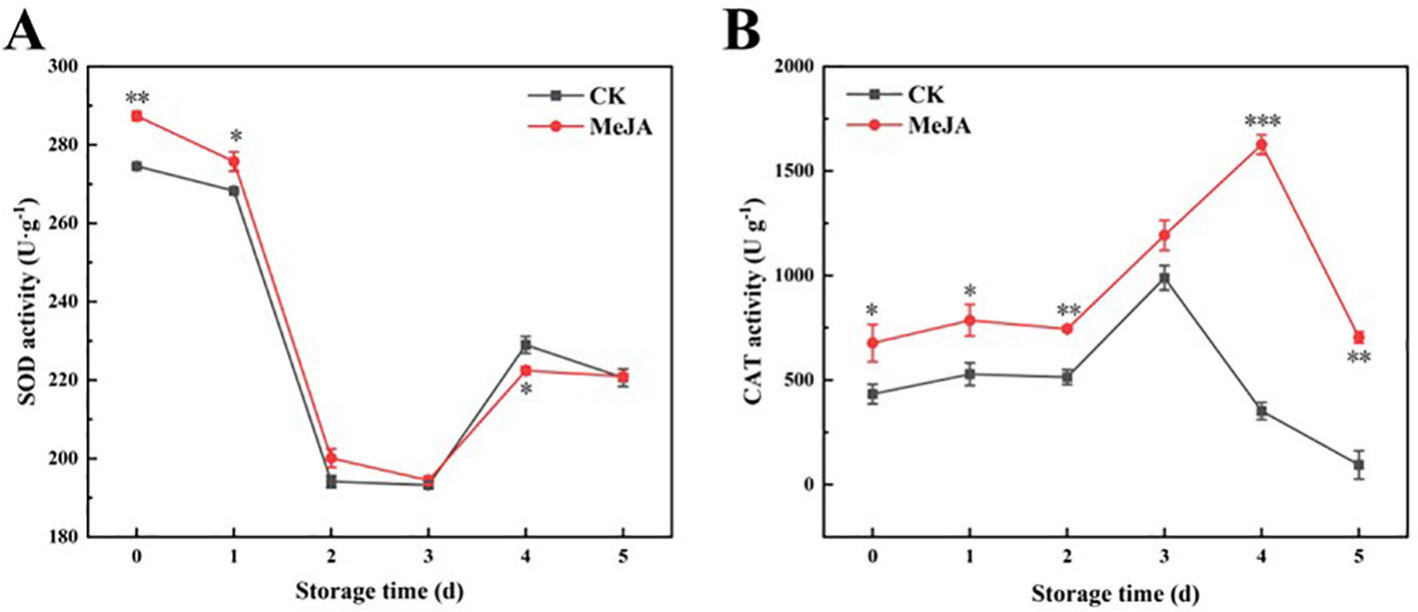
The effect of MeJA treatment on the activities of SOD **(A)** and CAT **(B)** of fresh-cut CWCs. Error bars represent the standard error of three biological replicates. *、**、***: represent the level of difference between MeJA group and CK group is *p* < 0.05、*p* < 0.01、*p* < 0.001, respectively.

Excessive ROS can lead to cellular damage and even apoptosis, while highly active antioxidant enzymes can mitigate the damage caused by ROS and maintain the relative balance of ROS metabolism. SOD catalyzes the conversion of O_2_·^-^ to H_2_O_2_, and CAT can convert H_2_O_2_ to O_2_ and H_2_O, helping maintain the cellular environment’s relative stability and protect cells from ROS (Kong et al., 2020). Both SOD and CAT activities exhibited a wave-like decreasing trend (**Figure 6**). SOD activity decreased sharply during the early storage stages, then increased and decreased slightly from day 3 to 5 (**Figure 6A**). Overall, MeJA enhanced the SOD activity of fresh-cut CWCs.

As shown in **Figure 6B**, CAT activity in the MeJA group was significantly higher than that in the CK group as a whole and peaked at day 4.In short, MeJA also improved the CAT activity of fresh-cut CWCs.

### 3.7 Gene expression levels

**Figure 7:**
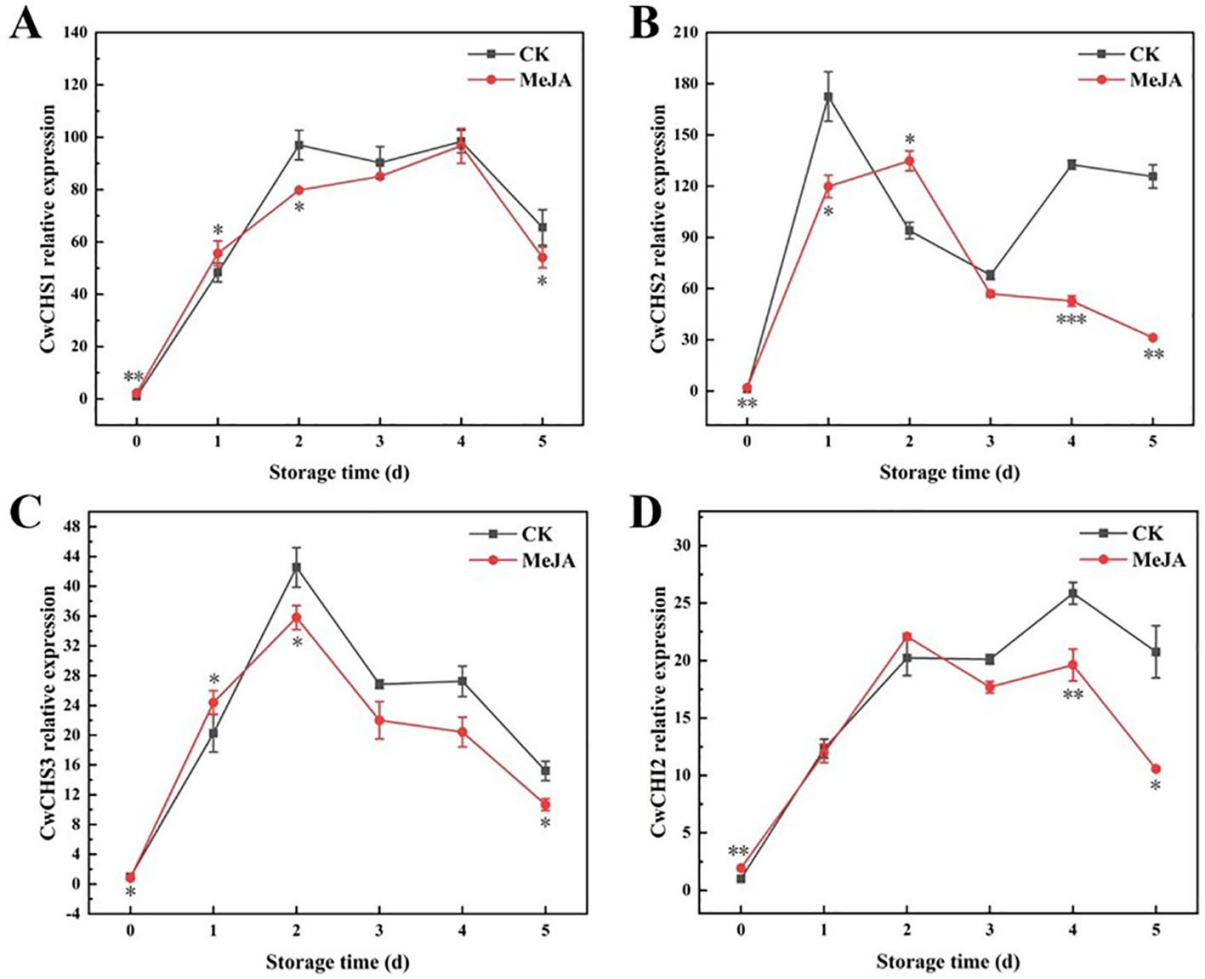
Gene expression of *CwCHS1* **(A)**, *CwCHS2* **(B)**, *CwCHS3* **(C)**, and *CwCHI2* **(D)** in MeJA-treated fresh-cut CWCs. *、**、***: represent the level of difference between MeJA group and CK group is *p* < 0.05、*p* < 0.01、*p* < 0.001, respectively.

In this experiment, in order to investigate the mechanism by which MeJA can delay surface discoloration and quality deterioration of fresh-cut CWCs during storage, *CwCHS* and *CwCHI2* genes were obtained for qPCR analysis using the materials of this experiment (fresh-cut CWCs). In the phenylpropane pathway, *CHS* and *CHI* are important precursors and key enzymes for flavonoid synthesis (Song et al., 2019). During the storage period, the expression levels of *CwCHS1* and *CwCHI2* genes showed an overall increasing trend, but were significantly lower in the MeJA-treated group compared with the CK group (**Figure 7**). As can be seen from **Figures 7A, C**, the MeJA group significantly down-regulated the expression of *CwCHS1* and *CwCHS3* genes in fresh-cut CWCs compared to the CK group (2–5 d). However, the expression level of *CwCHS2* gene was significantly higher in the CK group than in the MeJA group except for day 2, and the MeJA group significantly down-regulated the expression of *CwCHI2* gene in the later stages of storage (3–5 d). The *CwCHS2* and *CwCHI2* gene expression levels in the CK group were 5.02 and 2.97 times higher than those in the MeJA group (5 d), respectively (**Figures 7B, D**). The results indicated that MeJA inhibited the accumulation of flavonoids in fresh-cut CWCs probably by down-regulating the expression of *CwCHS1*, *CwCHS2*, *CwCHS3* and *CwCHI2*, which delayed the surface discoloration of fresh-cut CWCs.

### 4 Discussion

People generally assess the freshness of fruits and vegetables based on sensory qualities such as appearance, smell, texture, and taste (Xu et al., 2023a), a principle that especially applies to fresh-cut products. Due to mechanical damage and the loss of their protective outer layer after peeling, water chestnuts are prone to browning when exposed to air, significantly shortening their shelf life (Xu et al., 2022). Among our findings, the immersion of fresh-cut CWCs in 20 μM MeJA for 10 min notably suppressed yellowing, supported by the L*, a*, b* values and the degree of browning. MeJA treatment has also been observed to effectively inhibit browning in other produce, such as litchi fruit (Deshi et al., 2021), thereby corroborating our findings.

The enhanced respiration and transpiration in fresh-cut CWCs, resulting from loss of their outer skin protection, make them more vulnerable to water loss during storage. Mechanical damage compromises cellular integrity, accelerating the loss of internal nutrients and exacerbating weight loss. Soluble solids content has been acknowledged to be a vital quality indicator (Liu et al., 2023). O_2_ and CO_2_ content can reflect respiratory intensity to a certain extent (Chen et al., 2022). MeJA has previously been shown to attenuate respiration and delay the weight loss of pomegranates (Garcia-Pastor et al., 2020). Our study also found that MeJA inhibited the respiration of fresh-cut CWCs, reducing weight loss and suppressing changes in soluble solid content, consistent with findings from studies on MeJA-treated ‘Kinnow’ mandarins (Baswal et al., 2020), and jujubes (Dong et al., 2016).

The increase in PAL activity promotes the formation and accumulation of phenolic compounds (Pen and Jiang, 2004; Kong et al., 2021). Our study observed a concurrent rise in PAL activity and TPC in fresh-cut CWCs, consistent with existing literature (Xu et al., 2022). Enzymatic browning results in the increased soluble quinone content in plant tissues (Teng et al., 2020). Numerous studies have shown that the total flavonoid content of fresh-cut CWCs increases with progressing yellowing (Li et al., 2016). In our study, the soluble quinone content, TPC, and TFC of fresh-cut CWCs gradually increased, yet MeJA suppressed this trend, maintaining a significantly lower degree of yellowing in appearance compared to the control. Several studies have suggested that the reduction of relevant pigment substances in plants, including flavonoids, carotenoids, total phenolics, chlorophylls, and anthocyanins, is the primary cause of postharvest discoloration in certain fresh produce (Zhang et al., 2023b). Huang et al. (2022) indicated that combining malic acid and lycopene could effectively alleviate the reduction of anthocyanins, flavonoids, and phenols, resulting in a more vibrant litchi peel. Similarly, Zhang et al. (2023a) concluded that salicylic acid inhibits the degradation of pigments, resulting in Longan peel containing more flavonoids, total phenols, carotenoids, and other pigment substances, thereby maintaining stable peel color. To some extent, these studies support our findings, suggesting that the reduction of TPC and TFC can delay the yellowing of fresh-cut CWCs, and MeJA seems effective in this regard.

Song et al. (2019)showed that PPO and POD, as key enzymes in the oxidation of phenolics, are one of the main causes of browning degree in fresh-cut fruits and vegetables. Meanwhile, SOD as an antioxidant enzyme played a key role in delaying the quality decline of fresh-cut fruits and vegetables. PPO exhibits high activity in damaged fruits and vegetables, promoting the conversion of polyphenols to quinones. On the other hand, POD catalyzes the oxidative polymerization of phenolics and flavonoids in fruits and vegetables in the presence of H_2_O_2_, leading to browning (Liu et al., 2018a). Studies on eugenol emulsion inhibiting the yellowing of fresh-cut CWCs by reducing enzyme activities (PPO, POD, especially PAL) and decreasing phenolic and quinone formation have been documented (Teng et al., 2020). Cai et al. (2024) showed that MeJA-loaded biofilm treatment inhibited POD activity and MDA content in loquat fruits, thereby slowing down their quality deterioration, which is similar to the results of the present study. Chitosan coating has been reported to effectively inhibit the discoloration of fresh CWCs, mainly by reducing the oxygen supply to freshwater chestnuts and inhibiting PAL, PPO, and POD activities (Pen and Jiang, 2003). Zhang et al. (2022) found that Aurone inhibited the yellowing of CWCs by reducing POD activity. Our research observed that MeJA inhibited PAL, PPO, and POD activities, accompanied by a decrease in TPC, TFC, and soluble quinones. Song et al. (2019) showed that ferulic acid treatment of fresh-cut CWCs significantly down-regulated the expression levels of *CwCHI1*, *CwCHS1*, and *CwCHS2*, which delayed the cut surface yellowing of fresh-cut CWCs. In this study, we found that the expression of *CwCHS1*, *CwCHS2*, *CwCHS3* and *CwCHI2* was significantly down-regulated in MeJA-treated freshly cut CWCs (**Figure 7**). This is consistent with the results of Xu et al. (2023b). We hypothesized that *CwCHS1*, *CwCHS2* and *CwCHS3* might be the key genes involved in the regulation of flavonoid accumulation by MeJA treatment. And *CwCHI2* may play a role in the late storage stage, thus inhibiting flavonoid accumulation in fresh-cut CWCs and achieving the purpose of delaying the surface discoloration of fresh-cut CWCs.

Plants naturally produce some reactive oxygen species during normal physiological metabolism (You et al., 2012). For example, as oxygen enters plant cells through respiration, it receives electrons to produce O_2_·^-^, which then transforms into H_2_O_2_ and OH·^-^. The content of ROS is usually balanced by the antioxidant system (Kong et al., 2020). However, certain stresses lead to a significant generation of ROS, disrupting the ROS balance and resulting in cell membrane damage, accelerating fruit oxidation and quality deterioration (Wang et al., 2023). SOD can catalyze the transformation of O_2_·^-^ into H_2_O_2_, while CAT can convert H_2_O_2_ into O_2_ and H_2_O, reducing cell membrane damage (Liu et al., 2023). During storage, the production rate of OH·^-^and the content of H_2_O_2_ and MDA in fresh-cut CWCs increased continuously, but those in the MeJA group were consistently lower than in the control. The O_2_·^-^ generation rate trend differed from other substances, showing an increase followed by a decrease. When analyzed in conjunction with the results, it could be explained by the initial decrease and subsequent increase in SOD activity. MeJA increased SOD activity, facilitating the conversion of O_2_·^-^, resulting in a slower O_2_·^-^ generation rate in the MeJA group. The increase in CAT activity in the MeJA group promoted the decrease in H_2_O_2_ content. In summary, the reduction of ROS and the enhancement of antioxidant capacity mitigated the oxidative damage to cell membranes, consequently reducing the production of MDA in the MeJA group. MeJA has been reported to activate SOD and CAT activities to maintain the quality of produce, such as blueberries (Wang et al., 2019a), which aligns with our results.

## 5 Conclusions

In conclusion, immersing freshly cut CWCs in 20 μM MeJA for 10 minutes effectively delays surface yellowing, inhibits respiration, reduces weight loss, and maintains soluble solid content during storage. MeJA also reduces the accumulation of TPC, soluble quinones by suppressing PAL, PPO, and POD activities, and significantly slows down the generation rates of O_2_·^-^ and OH·^-^, reduces H_2_O_2_ and MDA content by increasing SOD and CAT activities. Also, MeJA inhibited the synthesis of flavonoids in fresh-cut CWCs by down-regulating the expression of genes related to the phenylpropane metabolic pathway (*CwCHS1*, *CwCHS2*, *CwCHS3* and *CwCHI2*). Ultimately, MeJA effectively retards surface yellowing and maintains the quality of fresh-cut CWCs by reducing flavonoid production and enhancing antioxidant capacity.

## Data Availability

The datasets presented in this study can be found in online repositories. The names of the repository/repositories and accession number(s) can be found in the article/**Supplementary Material.**
