# Supplementary material for: Mechanism of exogenous methyl jasmonate in regulating the quality of fresh-cut Chinese water chestnuts
Source: Front Plant Sci. 2024 Aug 15;15:1435066. doi: 10.3389/fpls.2024.1435066 (PMC11362587; doi:10.3389/fpls.2024.1435066)
Supplement: Supplementary file 1 [file DataSheet1.docx]

**Supplementary Table S1. Primers and DNA sequences used for Real-Time PCR analysis.**

| Gene | Login number | Forward primer | Reverse primer |
| --- | --- | --- | --- |
| *CwCHS1* | MG719239.1 | TACTACTTCCGCATCACAA | TACCTCCACCACAACAAT |
| *CwCHS2* | MG719242.1 | TCGAGCGCCCTTTATTCCAG | AAGGTGCCCATCTATTGCCC |
| *CwCHS3* | MG719243.1 | TCAAGGCAATCAAAGAGTG | TAACAGATGGACGGAGAC |
| *CwCHI2* | MG719241.1 | TTCACTTCCTCCAAATCAAGTA | TCCTCCTCTTCTTCCTCGTA |
| *CwActin* | MG742687.1 | TATTCCTTCACCACCACAG | CTCCTGCTCATAGTCAAGA |


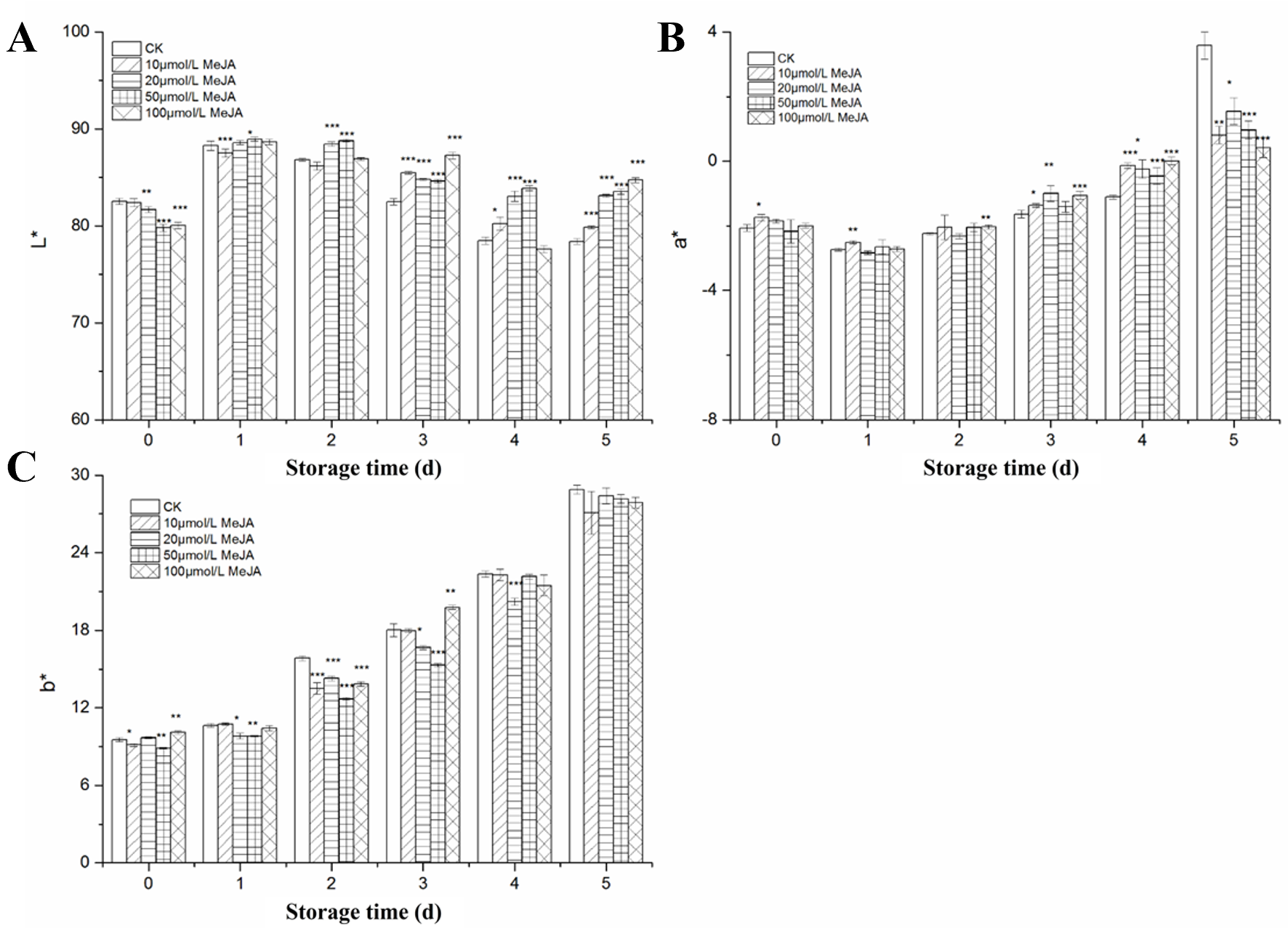


**Supplementary Figure 1.** The values of freshly peeled CWC L* (A), a* (B) and b* (C) treated with 10, 20, 50 and 100 μM MeJA. *、**、***: represent the level of difference between MeJA group and CK group is *p* < 0.05、*p* < 0.01、*p* < 0.001, respectively.
